# Personality Traits and Beliefs About Peers’ On-Road Behaviors as Predictors of Adolescents’ Moped-Riding Profiles

**DOI:** 10.3389/fpsyg.2018.02483

**Published:** 2018-12-07

**Authors:** Evelyn Gianfranchi, Mariaelena Tagliabue, Giulio Vidotto

**Affiliations:** Department of General Psychology, University of Padua, Padua, Italy

**Keywords:** adolescents, personality, beliefs, driving simulator, driving profiles

## Abstract

Several efforts aimed at discriminating between different degrees of on-road risky attitudes have been devoted to the identification of personality profiles among young drivers. However, the results are often inconsistent because of the limits of self-report measures. To overcome these limits, we tried to identify different profiles based on our study participants’ driving performances in a virtual environment and to look for psychological predictors of inclusion in one of three profiles. One-hundred and fourteen inexperienced adolescents were involved in this study, which included two experimental sessions. During the first, before riding along five virtual courses on a moped simulator, participants’ sensation seeking, locus of control, aggressiveness and beliefs about their peers’ on-road behaviors were measured by means of self-report tools. During the second session, the participants drove the simulator along six courses that were different from those faced in the first session. A cluster analysis was run on a wide number of indexes extracted from the participants’ performances to detect different riding profiles. Three profiles emerged (Imprudent, Prudent and Insecure), with specific riding patterns. The profiles also differed in terms of riding safety, assessed by means of the scores automatically given by the simulator to the participants’ performances. Reporting an external locus of control, underestimating peers’ on-road risky behaviors and showing less concern for fate among the possible causes of crashes are predictors that increase the risk of being included in the Imprudent profile. Low levels of dangerous thrill seeking predict inclusion in the Prudent profile, whereas high rates of self-reported anger play a role in discriminating the Insecure riders from the other profiles. The study indicates that it is possible to identify riding profiles with different degrees of on-road safety among inexperienced adolescents by means of simulated road environments. Moreover, inclusion in these profiles is predicted by different patterns of personality variables and beliefs. Further research is needed to verify the validity of these conclusions in real road conditions.

## Introduction

Road crashes were the major cause of death in adolescents worldwide in 2015, especially in males, whose mortality rates are consistently higher than those of females (World Health Organization [WHO], 2017). In 2014, road crashes were one of the main causes of death in Europe for people aged 15–19, representing 25% of the deaths at this age (Eurostat, 2017). Several efforts have been devoted to identifying the causes of this overrepresentation, resulting in a variety of explanatory models that include, among others, driving experience (Mayhew et al., 2003), hazard perception (Crundall, 2016), peers’ influence and adolescents’ beliefs about peers’ behavior (Allen and Brown, 2008) and personality traits (Arnett et al., 1997).

Mayhew et al. (2003) examined the month-to-month change in crash rate of adolescents, finding that the highest drop in the number of accidents occurred after 6 months of on-road experience. Some studies (Kinnear et al., 2013; Crundall, 2016) proved that driving experience is also linked to hazard perception, defined as the ability to predict dangerous on-road situations so as to act to prevent their negative outcomes (Tagliabue et al., 2017). Crundall (2016) verified that hazard prediction (*i.e*., the prediction of an imminent hazard) can discriminate between novice and experienced drivers. During three different experiments, participants watched video clips showing risky or safe on-road scenes, spotting for hazards (Crundall, 2016). The hazards could vary in terms of source, type and timing of the clues. Experienced drivers performed better than novice drivers across all three experiments, showing higher accuracy in spotting hazards and proving that hazard perception is modulated by different degrees of driving experience.

However, many other factors become important in shaping adolescents’ driving behavior, among which peer influence, beliefs about peers’ conduct, and personality traits have central roles.

### The Role of Beliefs and of Personality Traits in Adolescents’ Driving Behavior

Research has proven that the crash rate among adolescents rises consistently when they are with a peer (Preusser et al., 1998) and that teenagers tend to drive faster and to show more aberrant behaviors when carrying a peer than when carrying adults (Baxter et al., 1990). Baxter et al. (1990) claimed that these effects depend on both the tendency of teenage passengers to urge a driver to take risks (*e.g*., speeding or cutting a corner) and the need of teenage drivers to show off for their peer passengers. These behaviors are part of what Allen and Brown (2008) call direct (proximal) peer influence, which occurs when adolescents are driving and carrying their peers as passengers and which seems to affect drivers and passengers equally (Baxter et al., 1990; Ulleberg, 2004). Ulleberg (2004) examined the features affecting the likelihood of adolescents to ask their peers to drive safely when they feel unsafe as passengers. Overall, the results showed that young males are less prone to discouraging unsafe driving behaviors. Moreover, the majority of the sample, although reporting high rates of risky behaviors among their peers, found it acceptable to be their passengers. Peer influence can also be expressed indirectly (distal influence; Allen and Brown, 2008). Indeed, so-called “caravan peers” (*i.e*., peers driving other vehicles on the road), whose conduct is observed by adolescents, also have a role in influencing teenagers’ driving behavior (Allen and Brown, 2008). This indirect influence may shape adolescents’ norm setting and their beliefs about peers’ behavior and, in turn, it may lead to different degrees of risk in adolescents’ behaviors (either drivers or passengers).

For instance, about 11,000 adolescents in the United States participated in a survey on their beliefs about factors that affect driving safety (Ginsburg et al., 2008). More than a half of the respondents stated that they often or always see their peers involved in risky behaviors while driving, such as speeding or talking on the phone. However, only 15% of the respondents perceived the teenage drivers as inexperienced, although the 60% of the sample stated that inexperience heavily affects road safety. These results suggest that, although adolescents can detect risky driving behaviors among their peers, they do not perceive teenagers as inexperienced and as potentially dangerous drivers. Thus, beliefs about peers’ driving skills and behaviors may affect the development of adolescents’ defensive driving strategies (*e.g*., self-regulation on the basis of beliefs about others’ driving behaviors), contributing to the increased crash rate.

Among personality traits, sensation seeking (SS) is consistently linked to driving behavior. SS is usually defined as the tendency to seek novel, varied, exciting and intense sensations (Zuckerman, 1994). In a systematic review, Jonah (1997) found associations between high SS levels and risky driving in most of the examined articles. These associations were steady across cultures, stronger for males and tended to decline with age (Jonah, 1997). Overall, SS seems to account for up to 15% of variance in risky driving and, when the sub-dimensions of SS are considered, thrill seeking (TS) is the most related to on-road risky behaviors (Jonah, 1997). Among adolescents, high levels of SS are associated with driving while intoxicated, driving over the speed limit and racing other vehicles (Arnett et al., 1997). Moreover, SS predicts teenagers’ self-reported aggressive driving and driving anger (Dahlen et al., 2005).

Sensation seeking is frequently associated with aggressiveness in predicting reckless driving in adolescents (Arnett, 1996; Arnett et al., 1997; Ulleberg and Rundmo, 2003). Higher levels of aggressiveness (*i.e*., the tendency to act in a verbally and physically aggressive way and to experience anger and frustration) correspond to higher frequency of speeding behaviors among teenagers (Arnett, 1996; Arnett et al., 1997). Nevertheless, this relation might not be very clear. In a study that considered a variety of personality traits as possible predictors of self-reported risky driving behaviors, Ulleberg and Rundmo (2003) found only an indirect relationship between high aggressiveness and risky on-road behaviors, with a small-to-moderate effect size. The authors explained this result by claiming that personality traits in general may influence attitudes toward driving safety rather than the behavior itself. Another possible explanation may rely on the difficulty of assessing risky on-road behaviors with self-report measures. Moreover, the authors did not include in their model a trait that has been frequently reported as related to driving behaviors, *i.e*., locus of control (LC; Özkan and Lajunen, 2005), which may have a key role in moderating the relations between other personality variables and driving behaviors.

Özkan and Lajunen (2005) defined LC as a personality trait that reflects the degree to which people perceive events to be under their control or under the control of external forces that cannot be managed. The latter case is usually labeled “external LC” and it is associated with higher crash rates (Montag and Comrey, 1987). On the other hand, the results of Özkan and Lajunen (2005) showed a link between a more internal LC and higher number of self-reported crashes, violations and errors in a sample of young drivers. More recently, Warner et al. (2010) found a positive relation between internal LC and speeding behavior. A possible explanation of these results may be the involvement of overconfidence and of optimism bias (previously considered by Özkan and Lajunen, 2005). Indeed, drivers who think that their likelihood of incurring accidents depends only on their behaviors and skills may become overconfident and may develop fewer defensive driving strategies.

Given the inconsistency of some results, many studies have tried to identify profiles that combine specific personality traits and that can systematically account for risky driving behaviors.

### The Identification of Personality Profiles

The first approach adopted was to assess the relations between self-reported driving behaviors and drivers’ profiles that were identified through self-assessment personality measures. A survey of 6,000 Norwegian drivers, between 18 and 23 years old, was carried out by Ulleberg (2001). The author measured five personality traits (SS, anxiety, altruism, aggressiveness, and normlessness) and participants’ self-reported angry driving. In addition, several items were included to assess participants’ risky on-road attitudes and behaviors. A cluster analysis of the personality variables identified six groups. Two of them were considered at risk for road crashes: The first one was mostly composed of males and characterized by high levels of SS and normlessness but by low anxiety and altruism. The second at-risk cluster included participants with high scores in SS, anxiety, aggressiveness and angry driving. These two groups reported the riskiest driving habits and the highest frequency of road crashes and of harmful attitudes toward traffic (*e.g*., violating rules or speeding). The author concluded that, given the heterogeneity of the profiles’ characteristics and of their relations with self-reported risky driving behaviors and attitudes, young drivers cannot be treated as a homogenous group.

In Italy, Lucidi et al. (2010) detected different young drivers’ personality profiles and verified their relationship with self-reported aberrant on-road behaviors (Reason et al., 1990). The authors measured a wide number of personality traits (*e.g*., SS, anger, anxiety, and LC) and self-reported driving violations, errors, lapses and amount of accident involvement. Three clusters emerged: risky drivers (characterized by high levels of SS, angry driving and normlessness and by an external LC), worried drivers (high levels of anxiety and hostility) and careful drivers (high levels of altruism and low levels of anger, hostility, SS, and normlessness). The participants in the first group reported the highest crash rate and the riskiest driving attitudes while perceiving themselves as less prone to accidents. Careful drivers showed a reverse profile, reporting the lowest rates of errors, violations, lapses and crashes. Finally, worried drivers were classified as a medium-risk profile, because they reported better attitudes than risky drivers but also a comparable number of lapses.

These two studies proved that young drivers of different cultures can be grouped in clusters with specific personality patterns and that the personality profiles show different degrees of risky driving behaviors and attitudes as measured by self-report questionnaires. Moreover, the results by Lucidi et al. (2010) indirectly address the importance of drivers’ beliefs, showing that risky drivers may have less insight into their driving skills than both careful and worried drivers, overestimating themselves. However, the approach of these studies was based only on self-report measures, without a direct reference to behavioral variables.

Deery and Fildes (1999) tried to partially overcome the limits of self-report measures. First, they identified five clusters in a sample of adolescents (16–19 years old) on the basis of their personality traits and driving attitudes (*e.g*., hostility, assertiveness, SS, competitive speed and driving aggression). The most at risk cluster was characterized by, among others, high levels of hostility and of SS and by risky driving attitudes, such as high levels of competitive speed. Furthermore, participants in this cluster also reported high rates of risky driving behaviors but, at the same time, low crash rates. Then, the authors randomly selected a subsample of participants to test, through a driving simulator, whether the personality profiles differed in their behaviors during five courses with different features (*e.g*., driving while performing a calculation task, facing potentially hazardous scenes and facing an emergency situation). The results showed that the more at-risk cluster was also more prone to the negative effects of workload, had difficulties in facing the hazardous scenes, and was less cautious in terms of driving speed in the emergency situation. Overall, these results show that it is possible to identify different profiles among adolescent drivers and that the profiles differ in terms of personality patterns and attitudes toward risky driving. These differences were confirmed when the driving behavior was assessed by means of a simulator: the risky drivers had the least safe performance and showed a lack of hazard anticipation.

Marengo et al. (2012) considered fewer personality traits to identify different profiles among Italian adolescents (14–15 years old) with various degrees of moped-riding experience. Three clusters emerged: The so-called profile B showed high levels of SS and impulsivity and low levels of altruism and anxiety, being considered the most at-risk. Profile A was characterized by high levels of anxiety and low levels of SS and altruism. Profile C reported high levels of altruism and a more internal LC. Starting from the evidence that most of the previous studies used only self-report measures to assess the relation between the profiles and their driving behaviors (Ulleberg, 2001; Lucidi et al., 2010), Marengo et al. (2012) compared the clusters on the basis of self-report and simulated driving measures. Participants’ performances were assessed through 12 courses on a moped-riding simulator (Honda Riding Trainer, HRT), divided into three sessions. For each course, a letter score was provided: A (*safe performance*), B (*almost safe*), C (*near miss*), and D (*accident*). The first measure analyzed was the number of accidents (D score). In addition, the authors developed a safe driving index based on scores A, B, and C. The at-risk cluster showed the highest rate of self-reported risky driving behaviors (*e.g*., driving under the influence of substances and violations) and had the worst performance on the simulator, with the highest number of accidents and the lowest safe driving index score.

The main contribution of the study by Marengo et al. (2012) was its focus on adolescents, going beyond the limits of self-report measures, as Deery and Fildes (1999) suggested. The identified profiles were largely comparable to those that emerged in previous studies. For example, profile B was similar to the “risky drivers” in Lucidi et al. (2010), whereas profile A was comparable to one of the low-risk groups of Ulleberg’s (2001) study. The similarity between the teenagers’ clusters identified by Marengo et al. (2012) and previous results from samples with different ages suggests the presence of consistent differences also exists in adolescents in the early stage of driving experience.

### Simulator as a Tool to Assess Driving Profiles

The approach examined in the previous paragraph (the identification of different driving profiles on the basis of self-report measures of personality traits, driving attitudes and behaviors), albeit extremely useful, has three main limits: (1) self-report measures of driving attitudes and behaviors can be influenced by a number of biases (*e.g*., social desirability and overconfidence), preventing one from drawing predictions of real behaviors; (2) the use of these measures limits the inclusion of inexperienced drivers in the sample, resulting in the inability to discriminate between the role of driving experience and of personality traits in determining driving behaviors; and (3) the identification of profiles on the basis of personality traits led to inconsistent results, probably due to cultural peculiarities and to the instability of some personality traits during the lifespan (*e.g*., SS).

Driving simulators have been used to provide a behavioral correlate for the identification of driving profiles (Deery and Fildes, 1999; Marengo et al., 2012). An innovative approach was recently proposed by Gianfranchi et al. (2017a,b), aimed at identifying riding profiles on the basis of participants’ behavior on a moped-riding simulator. Reversing the approach of previous works, Gianfranchi et al. (2017a,b) monitored the performance of two samples of young drivers on five courses on the HRT simulator, measuring a wide number of variables (*e.g*., mean speed, mean pressure on the brakes, number of crashes and the overall performance evaluation) used to identify specific profiles. In the first study (Gianfranchi et al., 2017a), two clusters were identified (Imprudent and Prudent riders), with an opposite riding profile. Results showed that the two clusters also differed in terms of self-reported driving behaviors as measured by the Driver Behaviour Questionnaire (Reason et al., 1990) and the Dula Dangerous Driving Index (3DI; Dula and Ballard, 2003). For instance, Imprudent riders who answered the questionnaires after using the simulator (*i.e*., after having the chance to prove themselves in a series of potentially risky scenarios) reported lower rates of on-road errors and lapses, but they also reported a higher rate of on-road risky behaviors. In the second study (Gianfranchi et al., 2017b), a wider sample of young drivers was assessed by applying the same clustering procedure and measuring participants’ SS and non-contextual decision making through the Sensation Seeking Scale V (Zuckerman, 1994) and the Iowa Gambling Task (Bechara et al., 1994), respectively. Three clusters emerged: two of them resembled those already identified in the previous study, whereas the third showed mixed characteristics and was labeled “Insecure.” The results showed that the worst performance in terms of number of crashes and of overall performance evaluations was reached by participants with high levels of TS and poor decision-making ability.

These two studies were the first to adopt this procedure with the HRT simulator, which has already proved to be an effective tool for the enhancement of hazard perception among adolescents (Vidotto et al., 2011) and novice drivers (Tagliabue and Sarlo, 2015; Tagliabue et al., 2017), and this improvement is still present after 12 months (Vidotto et al., 2015). Among others, the roles of attention (Tagliabue et al., 2013), workload (Di Stasi et al., 2009), feedback (Megías et al., 2017), and of visual exploration (Di Stasi et al., 2011) in driving behaviors have been assessed through the HRT, adding important evidence to psychophysiological and cognitive models of driving behaviors. In respect to these previous findings, the results of the studies by Gianfranchi et al. (2017a,b) indicate that this simulator can be also used as an assessment tool, allowing the identification of different profiles based on a deep monitoring of a variety of driving variables. Moreover, the profiles have shown to be linked to self-reported driving behaviors, sensation seeking and decision making. However, none of these studies aimed at identifying predictors of the inclusion in the driving profiles, nor have they focused on totally inexperienced participants so as to isolate the role of personality or of cognitive predictors.

### Aims of the Study

Starting from the previous evidence, we speculated that because personality variables and beliefs have a central role in adolescents’ on-road behaviors (Arnett et al., 1997; Allen and Brown, 2008), they may be predictors of the inclusion in different riding profiles that can be identified by the HRT simulator. Thus, we reversed the methodology used by Marengo et al. (2012), using the simulator to test inexperienced participants and to identify potentially risky riding profiles that can be predicted by specific combinations of personality traits and beliefs. This approach would lead to the possibility of overcoming the limits of self-report driving behavior measures and of the problematic identification of personality profiles, allowing a direct link to be drawn between personality, beliefs and driving behaviors, with important preventive implications. Thus, the aims of the present study are (1) the identification of different profiles of simulated moped-riding in adolescents with no on-road experience and (2) the assessment of the relations between the driving profiles and personality traits and beliefs about their peers’ on-road risky behaviors.

For the first aim, we based our work on the methodology developed by Gianfranchi et al. (2017a,b) so as to test participants’ driving behaviors directly, even if inexperienced. Indeed, after a proper familiarization, we speculated that adolescents, although inexperienced, would show different degrees of risk while driving and that the differences in the identified profiles would depend not on experience but on other variables, such as personality traits and beliefs. The familiarization would allow to overcome the limits of the participants’ inexperience with the virtual environment and with the driving task in general. To do so, we decided to divide the procedure into two sessions: the first one was intended as a familiarization session, whereas the second was employed to test the participants’ driving behaviors.

For the second aim, we measured adolescents’ self-reported SS, LC, aggressiveness and beliefs, considering them as predictors of inclusion in the profiles. Beliefs were assessed through the 3DI questionnaire (Dula and Ballard, 2003). The original 3DI questionnaire does not assess the behavior of the peers. However, considering that participants could not answer to the items on the basis of their own driving experience (since they had no on-road experience), they were asked to rate the frequency of the behaviors described in the items among their peers. Indeed, although developed to assess experienced drivers’ dangerous actions, the 3DI items refer to behaviors that most people can judge as dangerous or inappropriate (*e.g*., “I will weave in and out of slower traffic” or “I verbally insult drivers who annoy me”), even without proper driving or riding experience.

## The Study

### Participants

One hundred and fourteen adolescents (mean age: 14.85; range: 13–19 years; 59 males) enrolled in high schools of Padua, Italy, took part in the study. All of them had no on-road driving or riding experience, but they all used bicycles (60% of participants declared they rode a bike several times a week or each day). All of the participants had correct or correct-to-normal vision. They were not paid for their participation. Written informed consent was obtained by all the participants and, for the participants under the age of 18, also by their parents. The project has been approved by the Ethical Committee for the Psychological Research of the University of Padova.

### Tools

#### The HRT Simulator

The HRT is a riding simulator that includes a Pentium 4 PC with a Windows XP operating system and an LCD monitor (1024 × 768 resolution) placed on a base connected to a chassis equipped with moped-like controls that allow a person to ride along virtual courses. A speaker is placed on each side of the monitor through which instructions are given on the path to follow, in addition to reproducing the acoustic effect of the moped engine and the traffic.

**FIGURE 1:**
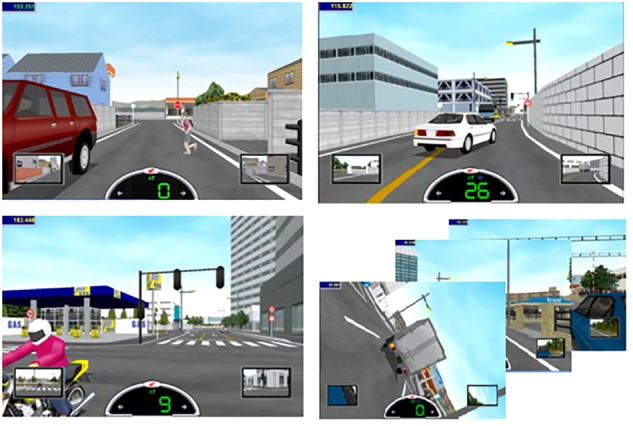
Examples of hazardous scenes classified on the basis of their risk degree: **(A)** (left top panel) and **(B)** scores (right top panel) vs. **(C)** (left bottom panel) and **(D)** (right bottom panel) scores.

The simulator provides a wide range of virtual courses, five on secondary roads and six on main roads. Each course includes seven or eight hazardous scenes [*i.e*., reconstructions of the most frequent hazardous on-road situations, based on the Maids Motorcycle Accidents In Depth Study (2004) classification]. The simulator gives a letter score for each scene, depending on how well a participant has prevented a crash (Figure 1). The scores can be A (*safe performance*), B (*almost safe*), C (*near miss*), and D (*crash*).

#### Questionnaires

All of the participants filled in a battery of questionnaires aimed at assessing their personality traits and their beliefs about peers’ on-road behaviors.

##### Sensation seeking

Sensation seeking was assessed through the Sensation Seeking Facets measure from the International Personality Item Pool (Hoyle et al., 2002), which includes 30 items divided into three subscales (10 items each) aimed at measuring different aspects of TS. Each subscale includes 10 items that, respectively, assess the seeking of dangerous activities (Dangerous TS; “I might enjoy a free fall from an airplane”), the tendency to be impulsive and unpredictable (Impulsive TS; “I am unpredictable, people never know what I am going to say”) and the willingness to take calculated risks or to face the most common fears (Calculated TS; “I would love to explore strange places”). The items are scored on a 5-point Likert scale ranging from 1 (*strongly agree*) to 5 (*strongly disagree*).

##### Locus of control

We assessed participants’ LC through two self-report measures. The first one is the driving locus of control scale of the Italian Cognitive Behavioral Assessment (CBA BG; Vidotto et al., 1995), which includes 27 items (*e.g*., “Even an experienced and prudent driver can cause a serious accident” and “Prudence does not matter to avoiding traffic accidents”) on a 5-point Likert scale (from *strongly agree* to *strongly disagree*).

The second measure is the multidimensional Traffic Locus of Control scale (T-LOC; Özkan and Lajunen, 2005), aimed at discriminating between different dimensions of on-road LC. It is composed of four subscales (Others, Self, Vehicles and Environment, and Fate) in which participants have to rate whether a crash can result from different types of circumstances (*e.g*., “Other drivers’ risk-taking,” “Bad weather or lighting conditions,” and “My own risk-taking”). The items are on a 5-point Likert scale (from *not at all possible* to *highly possible*).

##### Aggressiveness

The New–Buss questionnaire (N–B; Gidron et al., 2001), an eight-item self-report tool, was employed to assess participants’ aggressiveness. The questionnaire is the brief version of the Buss–Perry Aggression Questionnaire (Buss and Perry, 1992), and each of the four subscales that compose the tool includes two items of the original scale. The subscales are Verbal Aggression (“I can’t help getting into arguments when people disagree with me”), Anger (“Sometimes, I fly off the handle for no good reason”), Physical Aggression (“Given enough provocation, I may hit another person”), and Hostility (“I sometimes feel that people are laughing at me behind my back”). All the items are on a 5-point Likert scale (from *extremely uncharacteristic of me* to *extremely characteristic of me*).

##### Beliefs about peers’ on-road behaviors

Participants’ beliefs about peers’ on-road behaviors were assessed through the Dula Dangerous Driving Index (3DI – Dula and Ballard, 2003). The questionnaire includes 28 items divided into three subscales: Aggressive Driving (AD; 7 items; “I flash my headlights when I am annoyed by another driver”), Risky Driving (RD; 12 items; “I will drive if I am only mildly intoxicated or buzzed”), and Negative Emotions while driving (NE; 9 items; “When I get stuck in a traffic jam, I get very irritated”). Participants were asked to answer each item on a 5-point Likert scale from *never* to *always*, rating the occurrence of the on-road behaviors described by the sentences among their experienced peers.

### Procedure

The procedure included two experimental sessions that were scheduled a few days apart from each other. At the beginning of the first session, all of the participants filled in the questionnaires. Then, they were invited to sit on the HRT simulator, where an experimenter illustrated the riding controls and gave all the necessary information regarding the task. Participants were told to ride along the virtual paths as safely as they could, trying to avoid accidents. The HRT was set with moped controls, daylight conditions and automatic transmission so as to prevent any bias derived from riding inexperience.

During the first session, participants faced five courses on secondary roads, preceded by a practice course of 3 min during which they could explore the virtual environment and learn to use the controls. These five courses were introduced to allow participants (who were all inexperienced drivers) to familiarize with the virtual environment and the task. Six courses on main roads were faced during the second session: these courses were employed to test the presence of differences in terms of driving profiles among participants, after the familiarization phase (first session). Before starting the practice, all of the participants were asked about their knowledge on the main road rules and signals (*e.g*., traffic lights and stop signs), and all of them proved to be aware enough of the main rules and signals.

### Coding

Participants’ performances were constantly monitored through the HRT simulator, which collects a wide number of riding indexes with a sample rating of 30 Hz. As in previous works (Gianfranchi et al., 2017a,b), we extracted 18 indexes from participants’ performance in the second session. The indexes were mean and standard deviation of the throttle opening (%), the pressure on front and rear brakes (kg), on-road instability (horizontal deviations from the right side of the road), speed (km/h), number of braking, points on the path in which participants exceeded the speed limit, number of prevented accidents, time spent over the speed limit (in terms of number of frames), and mean and maximum over the limit speed value reached (km/h). Finally, a summary index (called Evaluation score) was extracted, based on the mean of the scores that the simulator automatically gave to the performance in each scene. The indexes were computed only on the courses of the second session. Indeed, we speculated that because our participants were all inexperienced, a proper riding profile could emerge only after familiarization with the virtual environment and the riding task. For the questionnaires, the original scoring instructions were followed.

### Design

The statistical analyses were divided into two main steps. After the inspection of the self-report measures (descriptive statistics, Cronbach’s alpha and correlations), the first main step was aimed at identifying the riding profiles among the participants in the second session through a cluster analysis. Then, we assessed differences between clusters in terms of risky behaviors through a multivariate analysis of variance (MANOVA) on the percentages of A, B, C, and D scores obtained during the second session, with *Cluster* as the between-participants factor. *Post hoc* analyses using Bonferroni’s correction were conducted, with α set at 0.05. Moreover, in order to rule out that the effects observed are due to differences in learning or driving skills already present before the test procedure in the second session, an identical MANOVA was carried out on the A, B, C, D scores of the first session (familiarization).

The second main step was aimed at identifying the psychological predictors of the inclusion in the riding profiles. Thus, we ran a multinomial logistic regression with the cluster solution as the dependent variable and the scores from the questionnaires as the predictors. All the analyses were performed with the IBM SPSS 23 statistical software package.

## Analysis and Results

As a preliminary step, descriptive and reliability statistics (Cronbach’s alpha) were calculated for the employed scales, along with Pearson’s correlations among them (Table 1).

**Table 1:** Pearson’s correlations, Cronbach’s alpha and descriptive statistics for the scales of the questionnaires.

		1	2	3	4	5	6	7	8	9	10	11	12	13	14	15	α	Mean (SD)	Range
**1**	Dangerous TS	-															0.81	26.1 (7.3)	11–50
**2**	Impulsive TS	0.73**	-														0.89	27.7 (8)	11–48
**3**	Calculated TS	0.57**	0.52**	-													0.69	36.7 (5.8)	22–50
**4**	CBA BG	0.39**	0.57**	0.24*	-												0.72	69.2 (9.6)	51–91
**5**	T – LOC Others	-0.18	-0.16	0.02	-0.22*	-											0.53	20.2 (2.1)	14–25
**6**	T – LOC Self	-0.11	-0.02	0.11	-0.17	0.59**	-										0.63	19.7 (2.6)	10–25
**7**	T – LOC VE	0.15	0.27**	0.21*	0.13	0.25**	0.13	-									0.55	15.5 (2)	10–20
**8**	T – LOC Fate	0.32**	0.45**	0.15	0.44**	-0.03	-0.07	0.30**	-								0.82	6.7 (2.6)	3–15
**9**	N-B verbal aggression	0.16	0.31**	0.14	0.33**	-0.09	-0.13	0.11	0.20*	-							0.41	5.4 (1.9)	2–10
**10**	N–B Anger	0.19*	0.40**	0.07	0.32**	-0.07	-0.01	0.10	0.16	0.59**	-						0.70	5.2 (2)	2–10
**11**	N-B physical aggression	0.26**	0.37**	0.25**	0.29**	-0.08	0.02	0.15	0.17	0.44**	0.53**	-					0.73	5.6 (2.3)	2–10
**12**	N-B Hostility	0.10	0.23*	0.01	0.16	0.04	-0.07	0.25**	0.07	0.41**	0.42**	0.30**	-				0.40	5.6 (1.8)	2–10
**13**	3DI AD	0.21*	0.22*	0.09	0.31**	-0.19*	-0.16	0.07	0.12	0.31**	0.31**	0.29**	0.14	-			0.74	12.9 (4.1)	7–32
**14**	3DI NE	0.09	0.27**	0.09	0.43**	0.01	0.08	0.11	0.10	0.43**	0.38**	0.31**	0.20*	0.64**	-		0.73	22.7 (5.1)	12–38
**15**	3DI RD	0.37**	0.39**	0.38**	0.48**	-0.22*	-0.14	0.05	0.18	0.27**	0.19*	0.26**	0.07	0.62**	0.52**	-	0.81	21.2 (6.6)	12–47

*Double and single asterisks indicate *p* < 0.01 and *p* < 0.05, respectively*.

The correlation coefficients show the presence of significant links among personality traits and between personality traits and beliefs. Cronbach’s alpha levels ranged from moderate (>0.50, for some of the scales with a low number of items) to high (>0.70) except for the subscales Verbal Aggression and Hostility of the N–B questionnaire. However, this last result is not surprising because the N–B scales include only two items each. Thus, following Briggs and Cheek’s (1986) suggestion, we calculated the inter-item correlations for each N–B scale. The coefficients are 0.26 for Verbal Aggression, 0.58 for Physical Aggression, 0.25 for Hostility, and 0.53 for Anger. Inter-item correlation coefficients higher than 0.20 are considered optimal (Briggs and Cheek, 1986).

**FIGURE 2:**
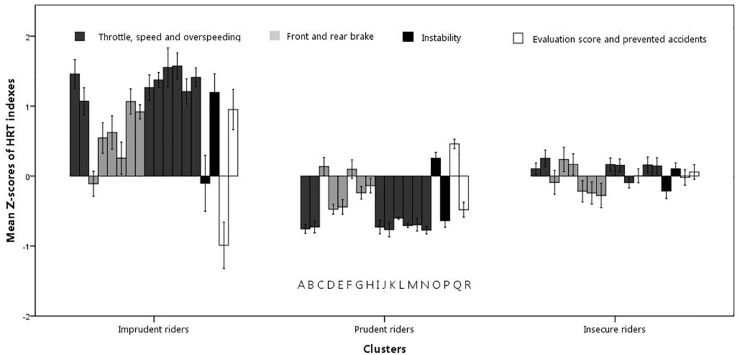
Mean *Z*-scores of the 18 HRT indexes in the three clusters. The indexes are listed in the order displayed by the letters on the bottom of the panel for each cluster. The indexes are the mean of the throttle opening (A) and its SD (B); number of times using the front brake (C); mean (D) and SD (E) of front brake pressure; number of times using the rear brake (F); mean (G) and SD (H) of rear brake pressure; mean (I) and SD of speed (J); time spent over the speed limit (K); number (L), mean (M), and the highest value (N) of speeding; mean (O) and standard deviation (P) of on-road instability; number of prevented accidents (Q); and mean Evaluation score (R; a higher score corresponds to a less safe performance). Vertical bars represent SE.

The next step was the identification of the riding profiles through a cluster analysis with the 18 HRT indexes of the second session used as grouping variables. The indexes were standardized (*Z*-scores) and analyzed with Ward’s method of hierarchical clustering with squared Euclidean distance measures. The inspection of the dendrogram and of the merging coefficients showed the presence of three clusters (profiles), with different riding patterns (Figure 2).

As depicted in Figure 2, the profiles report different trends on the riding indexes. The first profile, labeled “Imprudent” (21 participants; mean age: 14.90 years old; 15 males), showed a less safe behavioral pattern, with the highest values in almost all the riding indexes (*e.g*., speed, throttle opening, and Evaluation score). The second profile shows an opposite trend with respect to the Imprudent profile, with low values in all the riding indexes and high rates of prevented accidents. Thus, we labeled this profile “Prudent” (47 participants; mean age: 14.89 years old; 17 males). Finally, the third cluster, which in a previous work (Gianfranchi et al., 2017b) was labeled “Insecure,” shows a mixed pattern, with an overall safe performance, but with elements that can be potentially dangerous (*e.g*., tendency to exceed speed limits and, at the same time, hardly pressing on the front brake). This last cluster includes 46 participants (27 males) with a mean age of 14.78 years old. Although the profiles are homogenous in terms of age, a chi-squared test showed significant differences in terms of sex [*χ*^2^(2) = 8.71, *p* < 0.05]: females are predominant in the Prudent cluster (30 F vs. 17 M), whereas males are predominant in the Imprudent cluster (6 F vs. 15 M).

In order to better understand the differences among the identified riding profiles in terms of risky behaviors, a MANOVA was run on the percentages of A, B, C, and D scores of the second session (calculated over the total of the scenes) with the profiles as the between independent variable. At the multivariate level, the results show that the three profiles are significantly different with Wilks’ λ = 0.69, *F*(6, 218) = 7.43, *p* < 0.001, η_p_^2^ = 0.17. Univariate results indicate that significant differences are present in the percentages of each score, with *F*(2,111) = 16.64, *p* < 0.001, and η_p_^2^ = 0.23 for A score; *F*(2, 111) = 9.99, *p* < 0.001, and η_p_^2^ = 0.15 for B score; *F*(2, 111) = 6.58, *p* < 0.01, and η_p_^2^ = 0.11 for C score; and *F*(2, 111) = 17.74, *p* < 0.001, and η_p_^2^ = 0.24 for D score.

**FIGURE 3:**
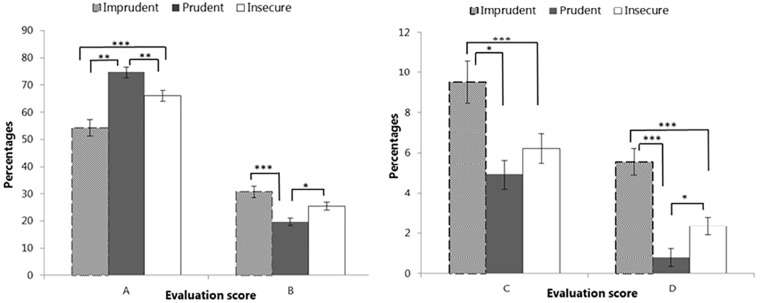
Differences in evaluation scores among the clusters. Vertical bars represent SE. Asterisks indicate significant differences in the *post hoc* comparisons with Bonferroni correction (^∗^*p* < 0.05, ^∗∗^*p* < 0.01, and ^∗∗∗^*p* < 0.001).

As depicted in Figure 3, Imprudent riders showed a less safe performance, with the lowest percentages of A scores (54.3%) than both Prudent (74.6%, *p* < 0.001) and Insecure riders (66%, *p* < 0.01). Imprudent riders showed also the highest percentages of C and D scores: C scores were 9.5% in Imprudent participants vs. 4.9% in Prudent (*p* < 0.001) and 6.2% in Insecure ones (*p* < 0.05); D scores were 5.6% in Imprudent riders vs. 0.8% in Prudent (*p* < 0.001) and 2.4% in Insecure riders (*p* < 0.001).

On the other hand, Insecure riders obtained lower percentages in A scores than Prudent riders (66 vs. 74.6%, *p* < 0.01) but higher than Imprudent riders (66 vs. 54.3%, *p* < 0.01), and higher D percentages than Prudent riders (2.4 vs. 0.8%, *p* < 0.05) but lower than Imprudent participants (2.4 vs. 5.6%, *p* < 0.001). Finally, they did not differ from Prudent riders in terms of C scores and from Imprudent riders in terms of B scores.

Overall, we can conclude that participants in the Imprudent cluster showed a less safe riding performance, with high percentages of scenes with crashes (D), near misses (C), and almost safe behaviors (B), reporting at the same time the lowest frequency of totally safe scenes (A). Prudent riders showed the opposite pattern, but they did not differ from Insecure riders in terms of near misses (C). Finally, participants in the Insecure cluster reported similar B percentages to those of the Imprudent cluster, testifying that Insecure riders’ performances, although overall better than those of Imprudent riders, included a significant amount of not totally safe scenes (*e.g*., hard braking or disrespecting safe distance).

As said, an identical MANOVA on the A, B, C, D scores obtained during the first session was carried out. Here, the factor *Cluster* failed to reach significance at the multivariate level (*p* = 0.111, η_p_^2^ = 0.06), thus allowing to rule out that the effects just described are due to differences in learning or driving skills already present before the test procedure (second session).

The last step of the statistical analysis consisted in a multinomial logistic regression (stepwise backward method) on the cluster solution as the dependent variable and the scores on all the questionnaires’ scales as predictors. The aim of the regression was to identify patterns of personality traits and beliefs that can predict inclusion in the riding profiles.

The final model was significant with *χ*^2^(18) = 44.99, *p* < 0.001, explaining 33% of the variance (Cox and Snell’s Pseudo *R*^2^ = 0.33) with a classification accuracy of 60.5%. Seven predictors reached significance in the final model (Table 2); that is, two dimensions of SS (Dangerous TS and Impulsive TS), two measures of locus of control (CBA BG and T-LOC Fate subscale), two dimensions of aggressiveness (N–B Anger and N–B Verbal Aggression), and beliefs about peers’ risky driving behaviors (3DI RD).

**Table 2:** Likelihood ratio test of the final regression model.

Likelihood ratio test			
	*χ*^2^	*Df*	*p*-value
*Intercept*	16.73	2	0.000
Dangerous TS	10.69	2	0.005
Impulsive TS	7.67	2	0.022
Calculated TS	5.35	2	0.069
CBA BG	8.58	2	0.014
T-LOC fate	8.91	2	0.012
N-B anger	7.75	2	0.021
N-B verbal aggression	7.29	2	0.026
3DI RD	6.40	2	0.041
3DI AD	5.00	2	0.082

The regression coefficients reported at the top of Table 3 show that the likelihood of being included among Imprudent riders with respect to Prudent and Insecure profiles was increased by lower scores on the 3DI Risky Driving scale (*p* < 0.05) and on the T-LOC Fate scale (*p* < 0.05 compared with Prudent riders and *p* < 0.01 compared with Insecure riders) but by higher scores at the CBA BG (*p* < 0.01 with respect to Prudent participants and *p* < 0.05 with respect to Insecure participants). Moreover, higher scores on the Dangerous TS and N–B Verbal Aggression scales play a significant role (*p* < 0.05) in discriminating between Imprudent and Prudent profiles.

**Table 3:** Parameter estimates of the regression with imprudent (top of the table) and insecure (bottom of the table) profiles as reference categories.

Prudent riders	Beta	*χ*^2^	*Df*	*p*-value	Insecure riders	Beta	*χ*^2^	*Df*	*p*-value
*Intercept*	*13*.*51*	*11*.*19*	*1*	*0*.*001*	*Intercept*	*12*.*94*	*10*.*55*	*1*	*0*.*001*
Dangerous TS	-0.18	6.48	1	0.011	Dangerous TS	-0.05	0.43	1	0.512
Impulsive TS	0.09	1.82	1	0.177	Impulsive TS	-0.06	0.69	1	0.406
Calculated TS	-0.11	2.01	1	0.157	Calculated TS	-0.17	4.81	1	0.028
CBA BG	-0.12	7.38	1	0.007	CBA BG	-0.10	4.83	1	0.028
T-LOC fate	0.30	3.87	1	0.049	T-LOC fate	0.42	7.58	1	0.006
N-B anger	0.14	0.51	1	0.475	N-B anger	0.50	5.71	1	0.017
N-B verbal aggression	-0.53	6.41	1	0.011	N-B verbal aggression	-0.37	3.28	1	0.070
3DI RD	0.17	4.61	1	0.032	3DI RD	0.18	4.79	1	0.029
3DI AD	-0.06	0.42	1	0.518	3DI AD	-0.20	3.79	1	0.052

**Imprudent riders**	**Beta**	***χ*^2^**	***Df***	***p*-value**	**Prudent riders**	**Beta**	***χ*^2^**	***Df***	***p*-value**

*Intercept*	-*12*.*94*	*10*.*55*	*1*	*0*.*001*	*Intercept*	*0*.*56*	*0*.*06*	*1*	*0*.*803*
Dangerous TS	0.05	0.43	1	0.512	Dangerous TS	-0.13	6.35	1	0.012
Impulsive TS	0.06	0.69	1	0.406	Impulsive TS	0.15	6.67	1	0.010
Calculated TS	0.17	4.81	1	0.028	Calculated TS	0.62	1.45	1	0.229
CBA BG	0.10	4.83	1	0.028	CBA BG	-0.02	0.46	1	0.499
T-LOC Fate	-0.42	7.58	1	0.006	T-LOC Fate	-0.12	1.29	1	0.257
N-B Anger	-0.50	5.71	1	0.017	N-B Anger	-0.36	4.33	1	0.038
N-B verbal aggression	0.37	3.28	1	0.070	N-B verbal aggression	-0.15	0.84	1	0.359
3DI RD	-0.18	4.79	1	0.029	3DI RD	-0.01	0.02	1	0.881
3DI AD	0.20	3.79	1	0.052	3DI AD	0.14	2.96	1	0.086

When the Insecure profile is used as the reference category (bottom of Table 3), the coefficients show that higher scores on the N–B Anger scale predict inclusion in the Insecure profile, with respect to the other two profiles (*p* < 0.05). Moreover, reporting high scores on the Dangerous thrill-seeking scale (*p* < 0.05) but, at the same time, low scores on the Impulsive thrill-seeking scale (*p* < 0.05) increased the risk of being included in the Insecure profile, with respect to the Prudent profile.

**FIGURE 4:**
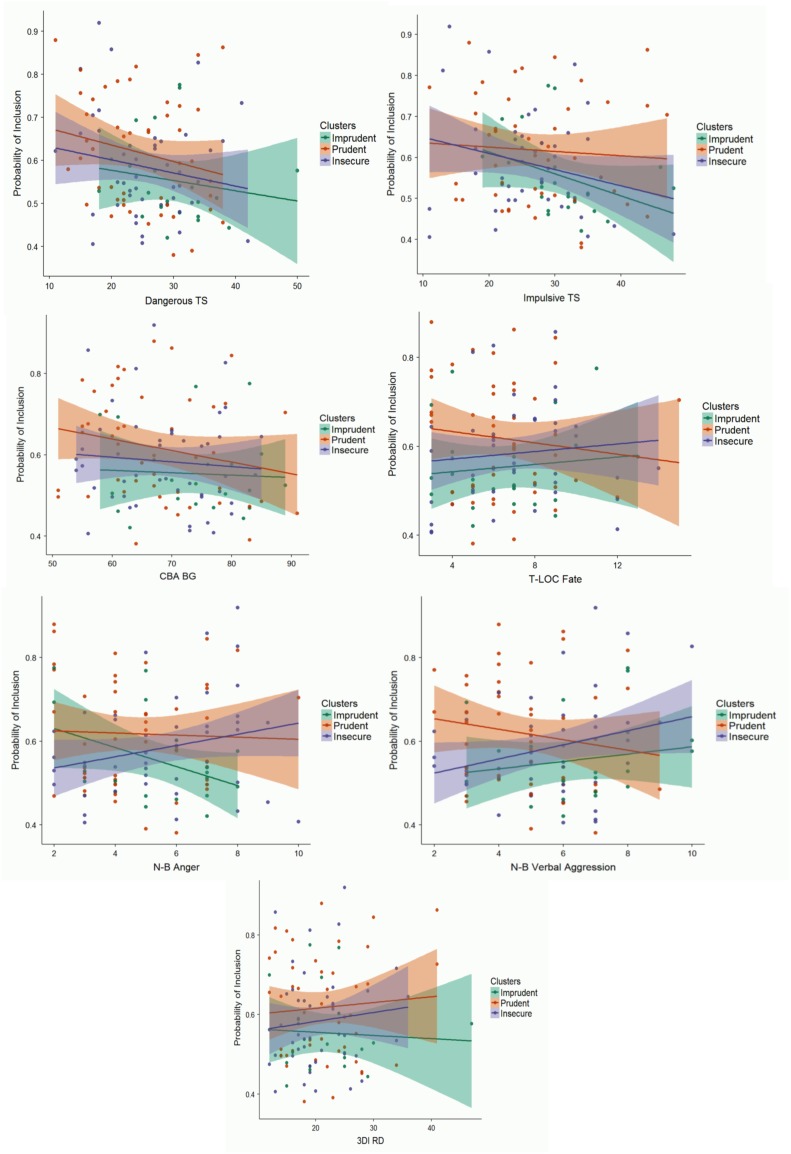
Probability of inclusion in the three profiles for the scores of each significant predictor. Shaded areas represent SE.

Overall, aspects such as an external locus of control, the underestimation of fate among the causes of crashes and of the frequency of peers’ on-road risky behaviors played a critical role in discriminating Imprudent riders from the other profiles (Figure 4). Moreover, participants with high levels of verbal aggression had a higher likelihood of being included in the Imprudent profile than in the Prudent profile. A low tendency to seek dangerous situations raised the probability of being included in the Prudent profile. Finally, inclusion in the Insecure profile was predicted by high levels of anger, whereas low levels of impulsivity played a role in discriminating between Insecure and Prudent riders.

## Discussion

This study has two main aims: the identification of different moped-riding profiles among inexperienced adolescents by means of a moped simulator and the assessment of the relations between the identified profiles and psychological predictors, such as sensation seeking, locus of control, aggressiveness and beliefs about peers’ on-road behaviors. The idea is to overcome the limits of previously employed methods, because the identification of a variety of drivers’ profiles based on self-reported personality traits and driving attitudes are rarely compared with objective driving indexes (in a real and in a simulated environment).

Following the procedure developed by Gianfranchi et al. (2017a,b), a cluster analysis was performed on 18 riding indexes of the second experimental session on the HRT simulator, allowing the identification of three moped-riding profiles in the present sample: Imprudent, Prudent and Insecure riders.

The profiles showed different riding patterns. The Imprudent riders exhibited the most unsafe pattern, with high speed and acceleration levels, high frequency of speeding behavior, and high rates of accidents and instability. The Prudent profile showed the opposite tendency, whereas the Insecure riders had intermediate characteristics. Moreover, a significant difference in terms of sex has emerged between the profiles. The Prudent profile is mostly composed of females, whereas the Imprudent profile is mostly composed of males. A number of studies (for a brief review see Oltedal and Rundmo, 2006) have proved that males are more prone to the effects of sensation seeking and to showing risky driving behaviors. This characteristic was also found in samples composed of adolescents (Oltedal and Rundmo, 2006; Marengo et al., 2012), and it seems to be present when a direct assessment of riding behaviors is performed, too.

Further analyses of the present data confirmed significant differences among the profiles in terms of risky behaviors. Indeed, Imprudent riders reported the lowest percentage of safe scenes and the highest percentage of near misses and crashes, whereas Prudent riders showed the opposite results. Insecure riders had overall a mid-range performance, with a percentage of near misses comparable to that of the Prudent profile but, at the same time, lower percentages of safe scenes and higher percentages of almost safe scenes; these last were comparable to those of the Imprudent profile.

Previous works (Lucidi et al., 2010; Marengo et al., 2012) identified three different profiles on the basis of self-report personality measures. In particular, Lucidi et al. (2010) detected three clusters (risky, worried, and careful drivers) that showed specific patterns of self-reported aberrant driving behaviors and risky attitudes, largely comparable to those showed on the HRT by the profiles in the present study. On the other hand, the three clusters identified by Marengo et al. (2012) in a sample of adolescents with various degrees of on-road experience, after being judged differently at-risk of road crashes on the basis of their personality traits, differed from each other in terms of riding safety on the HRT simulator. The present study, although confirming the results of previous studies, tries to go beyond them in three ways. First, it aimed to categorize different profiles based on a quantitative evaluation of their performance on the simulator. Second, it considered personality traits and beliefs as predictors of the profiles in an attempt to find a direct relation between them. Finally, the use of the questionnaire subscales allowed us to assess deeply, when present, the relation between personality traits, beliefs and riding performance.

Moreover, because this method has the advantage of allowing the direct assessment of participants’ driving behaviors and attitudes in a safe environment, it is also possible to test inexperienced road users to look for predictors of their performance. Indeed, contrary to previous studies (Gianfranchi et al., 2017a,b), here we decided to focus on totally inexperienced participants, so as to disentangle the role of on-road experience from that of other variables. Our results, besides identifying a cluster solution consistent with the one that emerged in a sample of novice drivers (Gianfranchi et al., 2017b), show that it is also possible to find inter-individual differences in driving behaviors among adolescents with no on-road experience, thus stressing the role of personality traits and beliefs.

As for the role of the predictors, our results are in line with the previous literature. Sensation seeking (especially dangerous TS) and locus of control seem to play key roles in the predictions of participants’ riding profiles, with high levels of SS and an external locus of control being associated with an increase in the risk of an imprudent behavior (Lucidi et al., 2010; Marengo et al., 2012). It is worth noting that lower scores on the Fate scale of the T-LOC predicted inclusion among Imprudent riders in our sample. Although attributing the causes of crashes to coincidence or fate may be interpreted as an index of external locus of control, at the same time also considering the role of unmanageable factors may have a role in developing defensive driving strategies, which in turn may lead to more cautious behavior.

Low levels of impulsivity and high levels of anger increased the risk of showing an insecure riding style among adolescents in our sample. Being less impulsive, although frequently associated with cautious behavior (Marengo et al., 2012), might also lead to difficulties in self-regulation of driving behaviors when a quick decision is required to face impending hazardous scenarios. This might explain how, in the present research, low levels of impulsivity are associated with insecure but not imprudent behaviors. However, further research is needed to support this conclusion.

Concerning the role of anger, Dahlen et al. (2012) tested a theoretical model of associations between different personality traits, aggressive driving and driving outcomes in a sample composed of adult drivers. Their results showed the existence of a positive relationship between low emotional stability (*i.e*., anger, depression, and anxiety) and aggressive driving, which in turn led to more on-road violations, near misses and crashes. In our sample, anger has proved to be predictive of the inclusion in the Insecure profile. At the same time, Insecure riders showed more reckless behaviors than Prudent riders, as attested by the lower frequency of safe scenes (A scores) and the higher frequency of both almost safe scenes (B scores) and crashes (D scores). These results are in line with the conclusions by Dahlen et al. (2012) as to road violations and crashes, indicating that higher levels of anger may represent a risk factor for less cautious driving behaviors. However, the result related to near misses has not been replicated. This discrepancy may be due to differences in age and experience of the involved samples or in the adopted questionnaires. Nevertheless, our study confirmed the key role of anger in predicting driving behaviors among adolescents.

Finally, underestimating peers’ on-road risky behaviors increased the risk of showing imprudent behavior on the HRT, with significantly higher percentages of crashes and near misses. Indeed, a correct estimation of others’ potentially hazardous behavior is crucial to preventing crashes and it represents the basis of the development of hazard perception and defensive driving strategies.

The principal limitation of the present study is related to the generalizability of the results to real on-road behaviors. Indeed, although it is true that the identification of profiles based on participants’ performances in a simulated environment rather than on self-report measures represents progress in the assessment methods of driving behaviors, there is still controversial evidence on the ecological validity of the simulators (de Winter et al., 2012). Thus, a further and necessary step will be following up on self-reported data and real on-road performance, especially focused on participants’ crash rates. Moreover, the application of the methodology reported in the present research to a sample of experienced adolescents (*i.e*., with at least 1 year of on-road experience) would offer the possibility to study the role of experience relative to that of personality traits.

Finally, a further limitation deals with the restricted battery of questionnaires used to assess personality variables. Indeed, risky driving is influenced by a number of variables, among which impulsivity or risk proneness play a prominent role (Megías et al., 2018). In addition, cognitive aspects (*e.g*., attention and decision making) are also thought to influence on-road behaviors (Tagliabue et al., 2013; Torres et al., 2017). Thus, further studies are needed to assess the role of other important personality traits and of cognitive predictors in determining the development of different driving and riding profiles.

## Conclusion

The present data indicate, first of all, that detecting different moped-riding profiles on the basis of a deep monitoring of the performance on a simulator is possible also among adolescents with no on-road experience. Second, the present study provides evidence that the identified profiles are not only dissimilar in terms of driving behaviors, but that they are also predicted by different personality patterns. These results represent the first step toward the development of an assessment method able to allow the early detection of risk-prone on-road profiles and of their predictors, along with potential protective factors. The practical implications of this new approach could range from the use of more complex virtual environments to identify driving profiles in specific populations with peculiar characteristics (*e.g*., older drivers or clinical populations) to the development of *ad hoc* training protocols that may provide a crucial contribution to preventing crashes.

## Ethics Statement

This study was carried out in accordance with the recommendations of guidelines for psychological research of the AIP – Associazione Italiana Psicologia with written informed consent from all subjects. All subjects, and the parents of the subjects aged less than 18 years old, gave written informed consent in accordance with the Declaration of Helsinki. The protocol was approved by the Ethical Committee for the Psychological Research of the University of Padua.

## Author Contributions

EG conducted data collection, statistical analysis, and manuscript writing. MT supervised data collection and contributed to statistical analysis and manuscript writing. All authors contributed to research planning, results discussion, and revision of the paper.

## Conflict of Interest Statement

The authors declare that the research was conducted in the absence of any commercial or financial relationships that could be construed as a potential conflict of interest.
